# Evaluation of protective immune responses induced by DNA vaccines encoding *Toxoplasma gondii* surface antigen 1 (SAG1) and 14-3-3 protein in BALB/c mice

**DOI:** 10.1186/1756-3305-5-273

**Published:** 2012-11-26

**Authors:** Min Meng, Shenyi He, Guanghui Zhao, Yang Bai, Huaiyu Zhou, Hua Cong, Gang Lu, Qunli Zhao, Xing-Quan Zhu

**Affiliations:** 1 Department of Parasitology, Shandong University School of Medicine, Jinan, Shandong Province, 250012, Peoples Republic of China; 2 State Key Laboratory of Veterinary Etiological Biology, Key Laboratory of Veterinary Parasitology of Gansu Province, Lanzhou Veterinary Research Institute, Chinese Academy of Agricultural Sciences, Lanzhou, Gansu Province, 730046, Peoples Republic of China; 3 College of Animal Science and Technology, Yunnan Agricultural University, Kunming, Yunnan Province, 650201, Peoples Republic of China

**Keywords:** *Toxoplasma gondii*, SAG1, 14-3-3, DNA vaccine, Immunity, BALB/c mice

## Abstract

**Background:**

Toxoplasmosis, caused by an obligate intracellular protozoan parasite *Toxoplasma gondii*, has been a serious clinical and veterinary problem. Effective DNA vaccines against *T. gondii* can prevent and control the spread of toxoplasmosis, which is important for both human health and the farming industry. The *T. gondii* 14-3-3 protein has been proved to be antigenic and immunogenic and was a potential vaccine candidate against toxoplasmosis. In this study, we evaluated the immune responses induced by recombinant plasmids encoding *T. gondii* surface antigen 1 (SAG1) and 14-3-3 protein by immunizing BALB/c mice intramuscularly.

**Methods:**

In the present study, BALB/c mice were randomly divided into five groups, including three experimental groups (pSAG1, p14-3-3 and pSAG1/14-3-3) and two control groups (PBS and pBudCE4.1), and were immunized intramuscularly three times. The levels of IgG antibodies and cytokine production in mouse sera were determined by enzyme-linked immunosorbent assays (ELISA). Two weeks after the last immunization, all mice were challenged intraperitoneally (i.p.) with 1×10^4^ tachyzoites of *T. gondii* and the survival time of mice was observed and recorded every day.

**Results:**

Mice vaccinated with pSAG1, p14-3-3 or pSAG1/14-3-3 developed high levels of IgG2a and gamma interferon (IFN-γ) and low levels of interleukin-4 (IL-4) and interleukin-10 (IL-10) compared to control groups (PBS or pBudCE4.1), which suggested a modulated Th1 type immune response (P<0.05). After intraperitoneal challenge with 1×10^4^ tachyzoites of *T. gondii* (RH strain), the survival time of mice in experimental groups was longer than control groups (P<0.05). Mouse immunized with pSAG1/14-3-3 induced a higher level of IgG antibody response and significantly prolonged the survival time when compared with pSAG1 or p14-3-3 (P<0.05).

**Conclusions:**

The study suggested that *T. gondii* 14-3-3 protein can induce effective immune responses in BALB/c mice and was a novel DNA vaccine candidate against toxoplasmosis, and the immune protective efficacy elicited by SAG1 gene was also demonstrated. Our results also showed multi-gene vaccine significantly enhanced immune responses and protective efficacy and was superior to the single-gene vaccine.

## Background

*Toxoplasma gondii* is a crescent shaped intracellular protozoan parasite that lives in various tissues of humans and other warm-blooded animals, causing toxoplasmosis [1]. Its life cycle is complex, including the sexual stage and the asexual stage; the sexual stage produces infective oocysts only in felines, whereas the asexual stage occurs in all infected animals and produces tachyzoites (the proliferative stage) and eventually, bradyzoites or zoitocysts (latent tissue cysts) [2,3]. There are three different infectious stages in the life cycle of *T. gondii*, i.e. tachyzoites, bradyzoites contained in tissue cysts and sporozoites inside sporulated oocysts, which are infectious for both intermediate and definitive hosts [4]. Epidemiological survey suggested that there was a wide distribution and high prevalence of *T. gondii* in many areas of the world and up to one third of the world’s population was infected [5-7]. In immunocompetent individuals, *T. gondii* infection is usually asymptomatic or solely causes mild symptoms; however, *T. gondii* can result in severe disease, such as ocular toxoplasmosis and encephalitis in immunocompromised patients and congenital birth defects [8,9]. Toxoplasmosis has been an important public health concern in some countries, especially in tropical countries, which leads to significant economic loss in human health and the farming industry [10].

Currently chemotherapy (a variety of antibiotics) is the primary strategy in the treatment of the acute phase of the disease and cannot act against chronic infection [11]. Due to adverse reactions and drug-resistance of *T. gondii* anti-parasitic drugs, there is an urgent need to develop effective measures for the control of *T. gondii*. Vaccination protocol has distinct advantages over chemotherapy and theoretically a single treatment can produce life-long protection; Therefore, there is a strong desire to develop vaccines against *T. gondii*[12]. During the past decade, anti-*T. gondii* vaccines have experienced different phases, containing live vaccines, attenuated-live vaccines, killed vaccines and subunit vaccines [13]. There is only a commercial vaccine for sheep, Toxovax, which is made of the live tachyzoites of strain S48, while there are none for humans [14-16]. In recent years, DNA vaccines against *T. gondii* have been well developed and received considerable attention and are an good option for this ubiquitous parasite [17]. A large number of experimental studies have shown DNA vaccines can elicit a predominantly Th1 immune response against toxoplasmosis in vaccinated mice; Furthermore, the DNA vaccines have the particular capacity to induce CD4+ T-lymphocyte and CD8+ cytotoxic T-lymphocyte (CTL) responses against the specialized antigen [18,19]. Proteins involved in the process of invasion of the host cell by *T. gondii* are regarded as the key candidate antigens in the design of the DNA vaccines. Thus, it is possible and promising to develop a safe and effective vaccine against human and animal toxoplasmosis.

14-3-3 proteins are a family of high sequence conservation and are present in all eukaryotic organisms, including plants, yeasts, protozoans, worms, insects and humans, which are involved in multiple pivotal biological regulatory processes, such as cell signal transduction, neuronal development and apoptosis [20,21]. In *T. gondii*, 14-3-3 protein was first found in the feline intraepithelial gametocyte stages [22]. Subsequently, it was detected in tachyzoites of *T. gondii* and presented in two different isoforms; The major isoform was cytoplasmic and to a lesser extent membrane-associated, whereas the minor one was associated with the detergent-resistant lipid rafts [23]. Meanwhile, the 14-3-3 protein was shown to be present in the parasitophorous vacuole (PV) of *T. gondii* tachyzoites and was proved to be a member of excreted secreted antigens (ESA); The recombinant 14-3-3 protein was immunogenic and played a vital role in stimulating the host immune system and the 14-3-3 protein was a potential vaccine candidate against toxoplasmosis [24]. In *Schistosoma mansoni*, it has been confirmed that 14-3-3 protein can induce significant humoral and cellular responses in mice, which provided moderate but significant protection against challenge infection [25]. However, the protective efficacy of 14-3-3 protein as a vaccine antigen against *T. gondii* remains unclear.

Although vaccines against toxoplasmosis are of different types, the recombinant subunit DNA vaccine has been promising with its strong immune responses in animal models and remaining cost effectiveness [26]. A range of studies suggested multi-antigenic DNA vaccines could provide significant protection against toxoplasmosis and are better than a single-gene vaccine [27,28]. As a good vaccine candidate, the main surface antigen of *T. gondii* tachyzoites, SAG1, is well described and is able to induce both effective and durable humoral and cellular immune responses in immunized mice [29-31]. In a word, SAG1 is a preferred antigen in constructing multi-gene DNA vaccine and is also regarded as a reference as compared to other antigens.

In this study, we constructed three eukaryotic plasmids, pBudCE4.1-SAG1, pBudCE4.1-14-3-3 and pBudCE4.1-SAG1/14-3-3, to examine humoral and cellular immune responses elicited by 14-3-3 and SAG1 protein in BALB/c mice. The survival time of mice after infection with the highly virulent RH strain of *T. gondii* was examined. The aim of this study was to evaluate the potential of *T. gondii* 14-3-3 protein as a vaccine candidate antigen, demonstrate the immune protective efficacy elicited by SAG1 gene and compare the immunological effect between single-gene and multi-gene vaccines.

## Methods

### Animals and parasite

Six to eight week-old female Kunming and BALB/c mice were purchased from Shandong University Laboratory Animal Centre (Jinan, China) and maintained under a standard specific-pathogen-free condition. In this study, Kunming mice were used to maintain and passage tachyzoites of *T. gondii*; BALB/c mice were used in the immunization experiment. All the animal experiments were approved by the Animal Ethics Committee of Shandong University.

The tachyzoites of *T. gondii* (RH strain) were collected from the peritoneal fluid of the infected Kunming mice. The peritoneal fluid was washed by 0.01M phosphate buffered saline (PBS) three times in a low speed centrifugation and disrupted using an Ultrasonic disintegrator, and then centrifuged at 2100×g for 15 min. The supernatant containing soluble tachyzoite antigens (STAg) was kept at −80°C until further use.

### Plasmid construction

The total DNA (or RNA) of *T. gondii* was extracted from purified tachyzoites according to the manufacturer's protocol. The open reading frame (ORF) of SAG1 gene [GenBank: S76248.1] and 14-3-3 gene [GenBank: AB012775.1] was obtained from *T. gondii* DNA or cDNA by PCR amplification using the following synthetic primers in which recognition sites were introduced and underlined.

SAG1: Forward primer: 5’-GAGTCGACATGTCGGTTTCGCTGCACCAC-3’ (Sal I)

Reverse primer: 5’-GCTCTAGATCACGCGACACAAGCTGCGAT-3’ (Xba I)

14-3-3: Forward primer: 5’-GCAGGTACCATGGCGGAGGAAATCA-3’ (Kpn I)

Reverse primer: 5’-GGCCTCGAGTTACTGATCAGCTTG-3’ (Xho I)

The PCR productions of SAG1 and 14-3-3 gene were respectively inserted into the pEASY-T1 Vector (TransGen, China) to generate two new prokaryotic plasmids. These recombinant plasmids were then transformed into *Escherichia coli* DH5α. After purification, pEASY-T1/SAG1 and pEASY-T1/14-3-3 were respectively double digested with appropriate restriction enzymes (Sal I/Xba I and Kpn I/Xho I) and purified from agarose gel. The pBudCE4.1 co-expression vector (Invitrogen, USA) is used to construct eukaryotic expression plasmids, which contains two promoters and is designed for simultaneous expression of two genes in mammalian cell lines. The SAG1 gene was inserted into pBudCE4.1 plasmid under the control of CMV promoter by the Sal I and Xba I cloning sites. The 14-3-3 gene was inserted into the Kpn I and Xho I sites of the EF-1α promoter in the pBudCE4.1. To construct multi-gene plasmid, the two genes were both inserted in pBudCE4.1 with appropriate cloning sites. The new plasmids were named pSAG1, p14-3-3 and pSAG1/14-3-3. The recombinant plasmids were transformed into *Escherichia coli* TOP10; the inserted genes were verified by PCR, restriction enzyme analysis and double-stranded sequencing. All the plasmids were purified using an endotoxin-free plasmid purification kit (TIANGEN, China). The concentration of purified plasmids was determined by spectrophotometer at OD 260 and OD 280, and the ratios were 1.8-2.0.

### Expression of recombinant plasmids *in vitro*

Hela cells were cultured in Dulbecco’s modified Eagle’s medium (DMEM, GIBCO) with 10% Fetal Bovine Serum (FBS), 100mg/ml streptomycin and 100 IU/ml penicillin at 37°C in 5% CO_2_. Before transfection, Hela cells were transferred in a 6-well plate (Costar, USA). When the density of hela cells reached 80%-90%, the recombinant eukaryotic expression plasmids (pSAG1, p14-3-3 and pSAG1/14-3-3) were transfected into cells using the Lipofectamine 2000 regent (Invitrogen, USA) according to the manufacturer’s guidance. The empty vector pBudCE4.1 was also transfected into Hela cells as a control. Lipofectamine 2000 reagent was respectively mixed with pSAG1, p14-3-3, pSAG1/14-3-3 and pBudCE4.1 at a concentration of 10μg/ml in DMEM without Fetal Bovine Serum (FBS) and antibiotics, and was incubated at room temperature for 20 min. The mixture of lipofectin and plasmid DNA was then added into Hela cells. The cells were incubated with the transfection mix for 6h at 37°C in the 5% CO_2_. At the end of incubation, fresh medium was supplemented and plates were returned to the cell incubator for further incubation. After 48h of transfection, Hela cells were collected and expression of the genes was evaluated by RT-PCR and Western blotting analysis.

Total RNA was extracted from the transfected Hela cells using Trizol Up (TransGen, China) and the mRNA transcription level of genes was identified by RT-PCR using specific primers of the inserted genes. RT-PCR products were then analyzed by agarose gel electrophoresis.

In addition, the transfected cells were treated on ice with RIPA Lyses Buffer (50mM Tris pH 7.4, 150mM NaCl, 1% Triton X-100, 1% Sodium deoxycholate, 0.1% SDS) containing 1mM protease inhibitor PMSF (phenylmethanesulfonyl fluoride) and centrifuged at 13,000×g for 5 min. The translation of genes in Hela cells was detected by Western blotting with anti-*T. gondii* polyclonal antibody (Goat) and a HRP (horseradish peroxides)-labeled rabbit anti-goat IgG antibody (Sigma, USA) as a secondary antibody. The conjugated substrate was detected using 3, 3’-Diaminobenzidine tetrahydrochloride reagent (Sigma, USA) as described previously [32,33] and pre-stained protein marker (Fermentas, Canada) was used as molecular mass standards.

### BALB/c mice immunization and challenge

To observe the immunogenicity of recombinant plasmids, BALB/c mice were randomly divided into five groups (12/group). Before vaccination, plasmids were diluted and suspended in sterile phosphate buffered saline (PBS) to a final concentration of 1μg/μl. All experimental groups were injected intramuscularly (i.m.) three times at weeks 0, 2 and 4 with plasmid DNA (100μg/each). Control groups received PBS (100μl/each) or empty plasmid (100μg/each). Blood samples of mice were collected from the tail vein plexus on the day before each vaccination and the sera were obtained and stored at −20°C for ELISA. Two weeks after the last injection, the mice in five groups were challenged intraperitoneally (i.p.) with 1×10^4^ tachyzoites of *T. gondii* RH strain as previously described [34] and the survival time of mice was observed and recorded every day.

### Determination of antibodies by ELISA

The levels of antibodies in mouse sera were determined by enzyme-linked immunosorbent assays (ELISA) as previously described [35]. Briefly, the microtiter plates (Costar, USA) were coated with 1μg STAg in 50mM carbonate buffer (pH 9.6) and incubated at 4°C overnight. After three washes, the plates were blocked with 1% Bovine Serum Albumin (BSA) for 1 h at 37°C and subsequently incubated with the mouse sera diluted in PBS for 1 h at 37°C. HRP-conjugated goat anti-mouse IgG, IgG1 or IgG2a (Sigma, USA) were used as the secondary antibody to detect bound antibodies. Finally, immune complexes were revealed by incubating with orthophenylene diamine (Sigma, USA) and 0.15% H_2_O_2_ for 30 min. The reaction was stopped by adding 2M H_2_SO_4_, and the absorbance was measured at 490 nm with an ELISA reader. All samples were run in triplicate.

### Cytokine assays

The levels of cytokines were detected according to the method described previously [36]. Two weeks after the final immunization, three mice per group were killed and the spleen was separated in a sterile condition. The spleen cells were cultured in 96-well plates at 37°C in 5% CO_2_ and cell-free supernatants were harvested and assayed for interleukin-4 (IL-4) at 24h, for interleukin-10 (IL-10) at 72h, gamma interferon (IFN-γ) at 96h using a commercial ELISA Kit (R&D Systems, USA) following the procedure recommended by the manufacturer.

### Statistical analysis

Statistical analysis in all groups was performed using SPSS software. The levels of cytokine and antibody production among different groups were analyzed and determined by one-way ANOVA. Survival time for the experimental and control mice was compared using the Kaplan-Meier method. The difference was considered statistically significant if P value was less than 0.05.

## Results

### Identification of the eukaryotic expression plasmids

**Figure 1:**
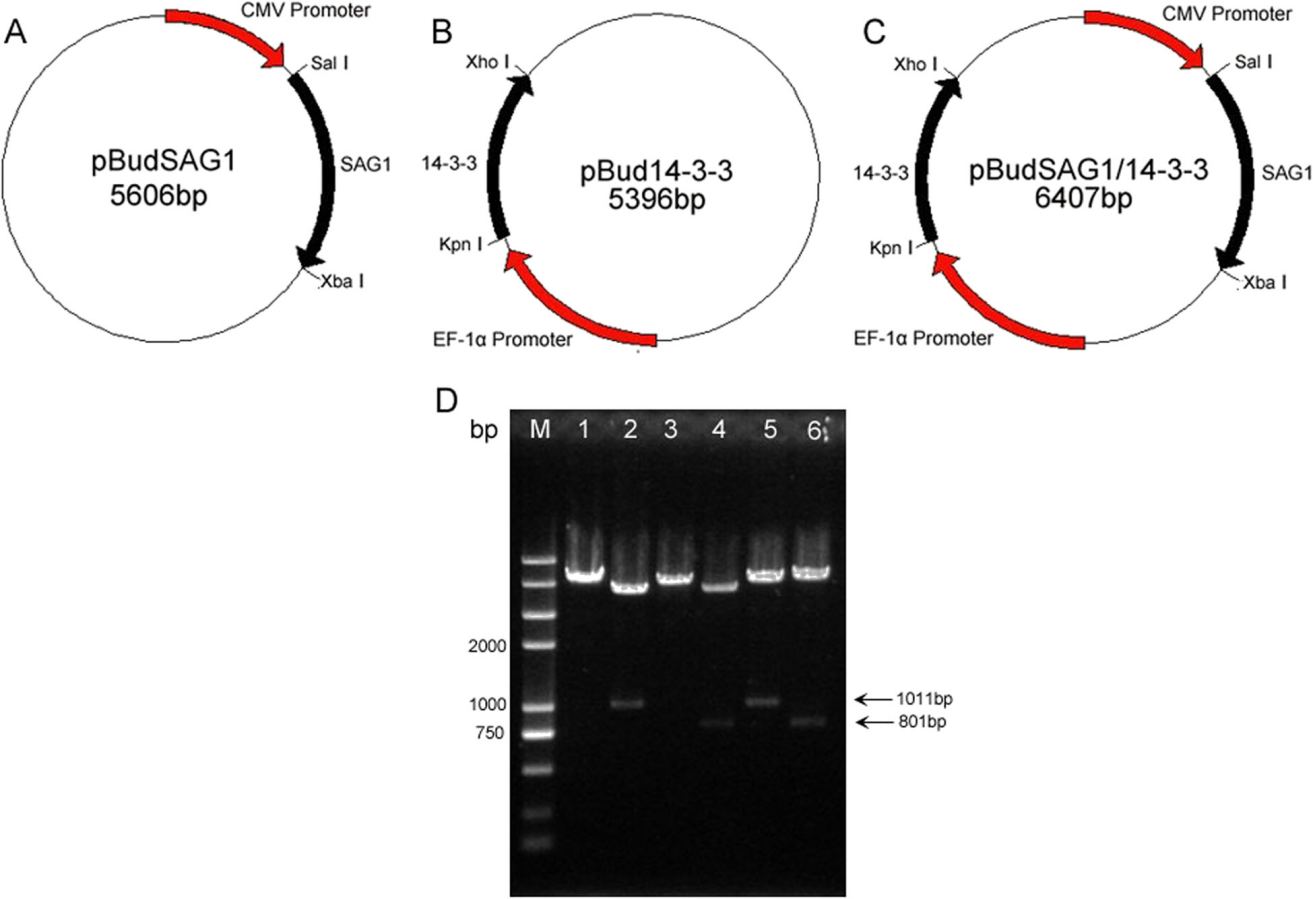
**Constructed plasmid maps and identification of the recombinant plasmids with restriction enzyme digestion.** (**A**) The SAG1 gene was inserted into pBudCE4.1 plasmid by the Sal I and Xba I cloning sites. (**B**) The 14-3-3 gene was inserted with the Kpn I and Xho I sites. (**C**) The two genes were both inserted in pBudCE4.1 with appropriate cloning sites. (**D**) DNA Mark (lane M), pSAG1 digested with Sal I (lane 1), pSAG1 digested with Sal I/Xba I (lane 2), p14-3-3 digested with Kpn I (lane 3), p14-3-3 digested with Kpn I/Xho I (lane 4), pSAG1/14-3-3 digested with Sal I /Xba I (lane 5), pSAG1/14-3-3 digested with Kpn I/Xho I (lane 6).

The SAG1 and 14-3-3 genes were cloned into the eukaryotic expression vector pBudCE4.1 through appropriate restriction enzymes to form three different plasmids (Figure 1A, B&C). To ensure the fidelity of plasmids, they were detected by PCR and restriction enzyme analysis, and confirmed by sequencing in both directions. The result of restriction enzyme digestion of plasmids is shown in Figure 1D.

### Gene expression in Hela cells

**Figure 2:**
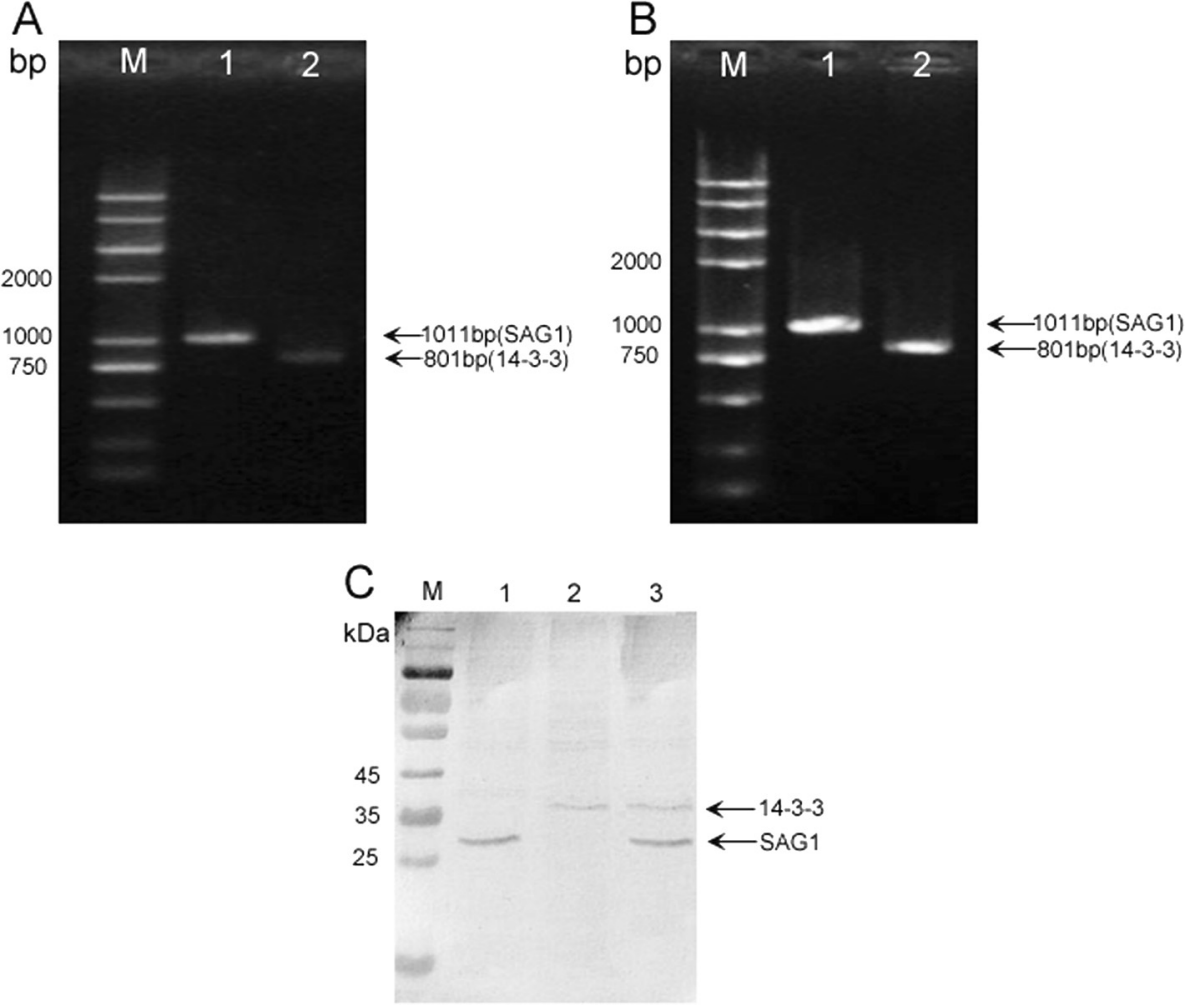
***In vitro *expression analysis of the constructs in Hela cells by RT-PCR and Western blotting.** (**A**) RT-PCR results for SAG1 and 14-3-3 (lane1 and lane 2) in cells transfected with pSAG1 or p14-3-3. (**B**) RT-PCR results for SAG1 and 14-3-3 (lane1 and lane 2) in cells transfected with pSAG1/14-3-3. (**C**) Pre-stained protein marker (lane M), hela cells transfected with pSAG1 (lane 1), cells transfected with p14-3-3 (lane 2), cells transfected with pSAG1/14-3-3 (lane 3).

Hela cells were transfected with pSAG1, p14-3-3 or pSAG1/14-3-3 for 48h, the level of mRNA and gene expression was evaluated by RT-PCR and Western blotting, respectively. Electrophoresis of RT-PCR products from transfected Hela cells showed the expected fragment of the SAG1 gene (1011bp) or 14-3-3 gene (801bp) (Figure 2A&B), which indicated that the transcriptional expression of the constructs was successful *in vitro*. In the Western blotting analysis (Figure 2C), the expression of SAG1 gene (about 30kDa) and 14-3-3 protein (about 36kDa) was respectively detected in Hela cells transfected with pSAG1 or p14-3-3; These two kinds of protein were both detected in cells transfected with pSAG1/14-3-3, whereas the empty vector transfected cells did not show any band. The results indicated that SAG1 and 14-3-3 proteins were successfully expressed and secreted by Hela cells and possessed immunological activity.

### Antibody responses in immunized BALB/c mice

**Figure 3:**
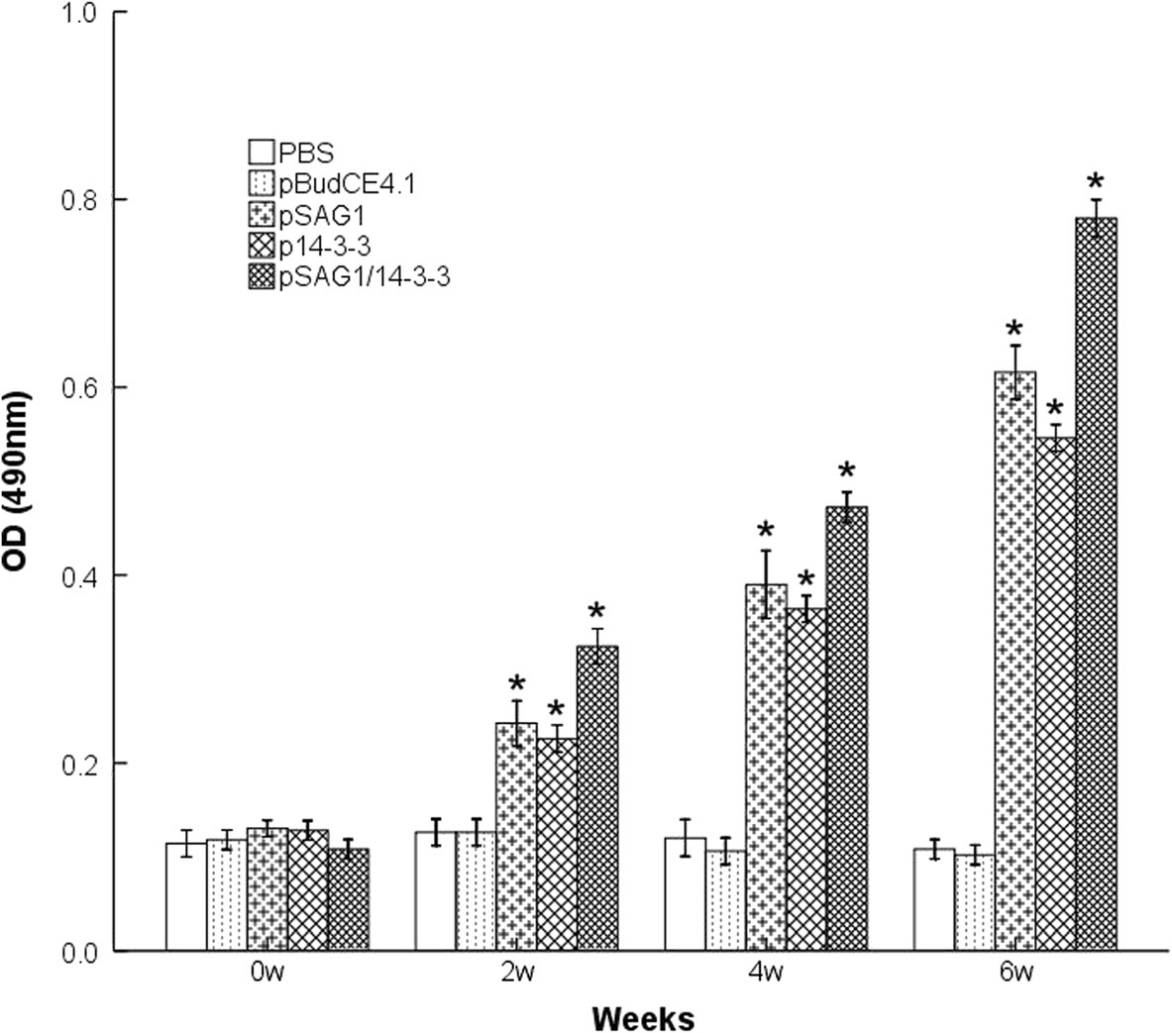
**Measurement of specific IgG antibodies in sera of immunized mice.** Sera were collected one day prior to each immunization and determined by ELISA. Results are shown as means of OD 490±SD and statistical differences (P<0.05) are indicated by * compared with PBS or pBudCE4.1.

The levels of IgG antibodies induced by recombinant plasmids in mice were detected post immunization by ELISA at weeks 0, 2, 4 and 6. As shown in Figure 3, significantly high levels of IgG antibodies were observed in the experimental group vaccinated with pSAG1, p14-3-3 or pSAG1/14-3-3 and the levels of antibodies gradually increased with successive immunization, which were higher than those of the control groups with PBS or pBudCE4.1. There was significant difference between the experimental groups and the control groups (P<0.05). The level of IgG antibody in pSAG1/14-3-3 group was higher than those of pSAG1 or p14-3-3 group (P<0.05). However, there was no statistical difference between the pSAG1 group and the p14-3-3 group (P>0.05). These results indicated that a recombinant plasmid encoding *T. gondii* 14-3-3 protein induced a strong IgG antibody response in mice, and OD value reached a high level two weeks after the third immunization.

**Figure 4:**
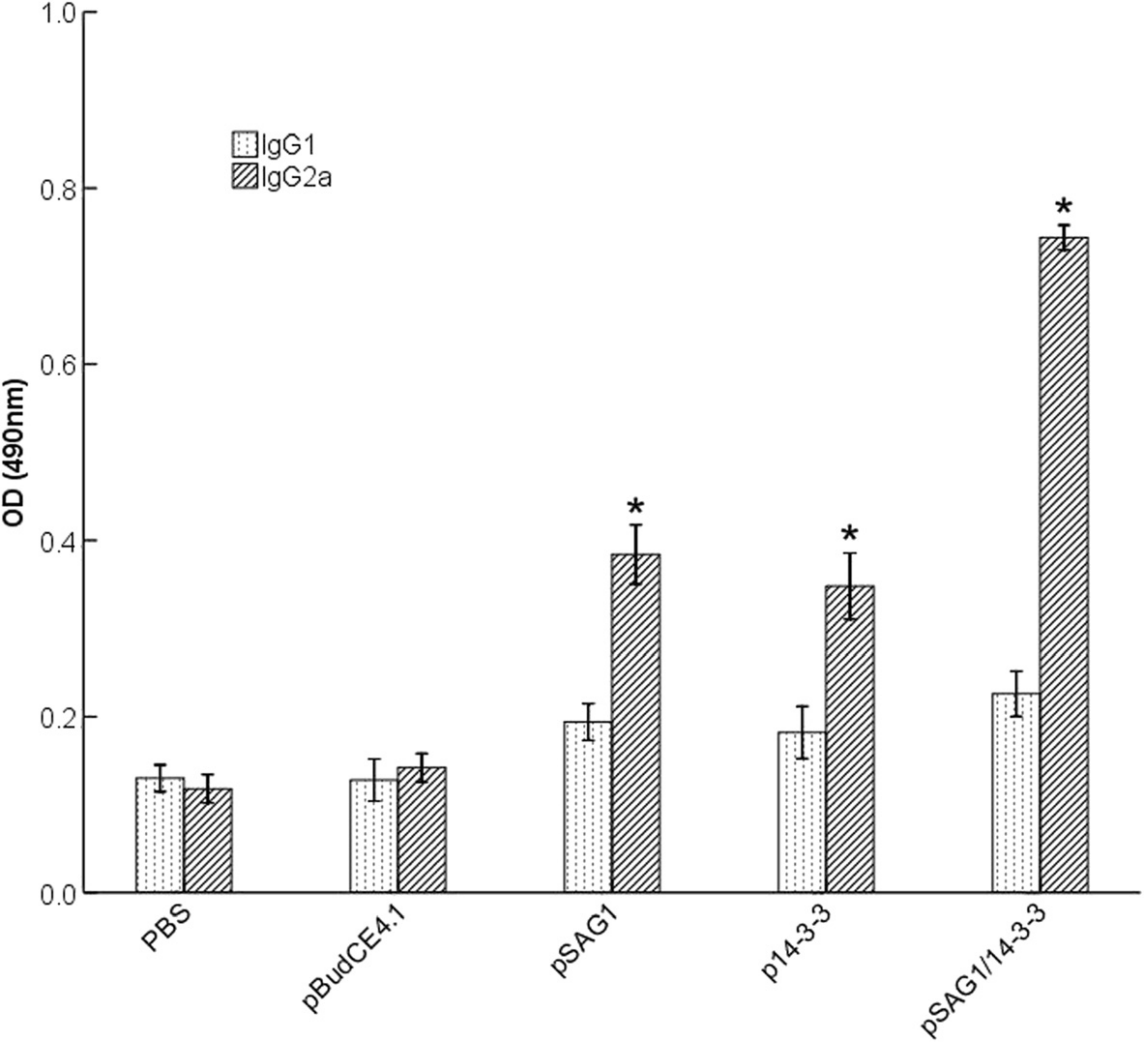
**Distribution of IgG subtypes IgG1 and IgG2a in immunized mice.** The levels of IgG subtypes IgG1 and IgG2a in sera of mice 2 weeks after the last immunization were analyzed by ELISA. Results are expressed as means of the OD_490±SD and statistically significant differences (P < 0.05) are indicated by an asterisk (*) as compared to control groups.

The levels of IgG subclass (IgG1 and IgG2a) in all groups at the second week after the final immunization were analyzed to determine whether a Th1 or Th2 response was elicited and the results are shown in Figure 4. An apparent predominance of IgG2a over IgG1 was observed in single-gene or multi-gene vaccine immunized mice (pSAG1, p14-3-3 or pSAG1/14-3-3), which indicated a shift toward the Th1 type response. Furthermore, mice immunized with pSAG1/14-3-3 generated higher levels of IgG2a than with pSAG1 or p14-3-3 (P<0.05). However, there was no significant difference in IgG2a levels between the groups immunized with pSAG1 and p14-3-3 (P>0.05). The data showed that vaccination with p14-3-3 in mice generated a Th1 immune response.

### Cytokine production

The supernatant of splenocytes was harvested at different times and used to measure the amounts of cytokine production (IFN-γ, IL-4 and IL-10) in different groups. As shown in Table 1, mice vaccinated with pSAG1/14-3-3 generated significantly higher levels of IFN-γ as compared to mice with single-gene plasmids, PBS or empty vector (P<0.05). The level of IFN-γ in pSAG1 immunized mice was higher than the p14-3-3, but there was no significant statistical difference between the two groups (P>0.05). Meanwhile, the low levels of IL-4 and IL-10 in the experiment and control groups suggested that there was no statistical difference among all the groups (P>0.05). Generally, IFN-γ and IL-2 favor Th1 type immune responses, whereas IL-4 and IL-10 favor the Th2 type [37]. With this knowledge, these results confirmed that the cellular immune response induced by single-gene or multi-gene vaccines ((pSAG1, p14-3-3 or pSAG1/14-3-3)) was inclined to a Th1 type response in mice.

**Table 1:** **Cytokine production by splenocyte**^**a**^** cultures from immunized BALB/c mice**

**Group**	**Production of cytokine(pg/ml)**^**b**^		
	**IFN-γ**	**IL-4**	**IL-10**
PBS	50.41±2.20	39.15±2.65	46.43±2.93
pBudCE4.1	50.80±1.75	35.68±2.88	40.87±2.82
pSAG1	698.14±88.65^*^	35.31±3.48	36.90±1.17
p14-3-3	605.53±108.83^*^	34.68±3.34	37.74±2.20
pSAG1/14-3-3	1188.23±169.15^*#^	38.83±3.02	37.23±1.33

^a^ Splenocytes from 3 mice per group two weeks after the final immunization.

^b^ Values for IFN-γ at 96h, IL-4 at 24h, IL-10 at 72h are expressed as mean ± SD.

^*^ Compared with PBS or pBudCE4.1 group, P< 0.05; #: compared with pSAG1 or p14-3-3, P<0.05.

### Survival time analysis against a lethal challenge in BALB/c mice

**Figure 5:**
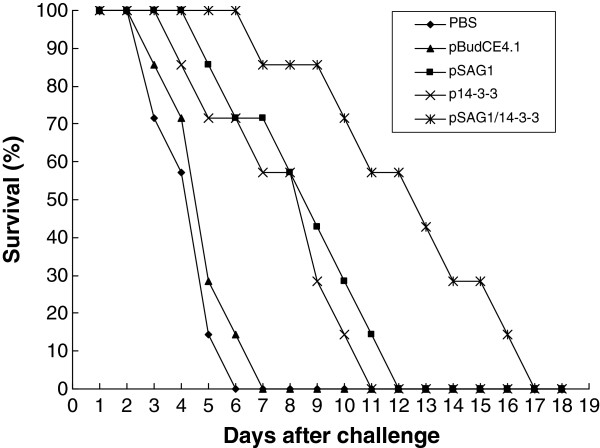
**Survival curves of vaccinated BALB/c mice against *T. gondii *infection.** The mice in five groups were challenged with 1×10^4^ tachyzoites of virulent *T. gondii* RH strain 2 weeks after the last immunization. Each group was composed of seven mice. Survival was monitored daily for 18 days after challenge.

To evaluate the immunoprotection induced by the DNA vaccines (single or multiple gene), all the mice were challenged intraperitoneally (i.p.) with 1×10^4^ tachyzoites of *T. gondii* RH strain and mortality was checked daily until all mice died. Survival percentage of mice in different groups is shown in Figure 5. Immunization of mice with the DNA vaccines (pSAG1, p14-3-3 and pSAG1/14-3-3) dramatically increased the survival time in comparison with the control groups vaccinated with PBS or pBudCE4.1 (P<0.05). Mice vaccinated with pSAG1/14-3-3 showed an increased survival time (11.5±3.5 days) compared to pSAG1 group (7.7±2.5 days) or p14-3-3 group (6.8±2.6 days) (P<0.05). However, no significant difference was observed between the groups of mice immunized with pSAG1 and p14-3-3 (P>0.05). All the experimental mice could not resist lethal acute *T. gondii* infections and died, which indicated that the DNA vaccine did not provide complete protection against *T. gondii*.

## Discussion

The present study showed that all mice in the experimental groups injected with pSAG1, p14-3-3 or pSAG1/14-3-3 induced the Th1 mediated immune responses, defined by high levels of IgG2a and IFN-γ, and low levels of IL-4 and IL-10. Significantly higher levels of IgG2a and IFN-γ were observed in mice immunized with pSAG1, p14-3-3 or pSAG1/14-3-3 as compared to those of controls and this increased with successive immunization. IFN-γ is the key mediator of resistance to *T. gondii* and promotes multiple complex intracellular mechanisms to kill the parasite and inhibit its replication [38]. Moreover, IFN-γ plays an important role in protecting hosts during both acute and chronic phases of toxoplasmosis [39,40]. Although IFN-γ is essential for long-term resistance to *T. gondii*, its overproduction can also induce severe immunopathology in animal models. It was reported that genetically susceptible C57BL/6 mice developed necrosis of the villi and mucosal cells in small intestines following peroral infection with *T. gondii* and that IFN-γ-mediated pathology in the small intestine predisposes to death in these mice; In contrast, IFN-γ was required for survival in infected genetically resistant BALB/c mice [41]. It appears that overproduction of IFN-γ occurs in the genetically susceptible mice following *T. gondii* infection and contributes to early death of these mice [41,42]. Thus, constructed DNA vaccines generated not only the protective effects but also possible severe immunopathology in animal models. When we evaluated the protective effect of vaccines in the genetically susceptible mice, mortality in mice caused by IFN-γ should be observed. In addition, in the design of DNA vaccines, potential immunopathology generated by proinflammatory mediators including IL-12, IL-18, IFN-γ, TNF-α and NO should be carefully considered.

Prolonged survival time after intraperitoneally challenge with 1×10^4^ tachyzoites of the virulent RH *T. gondii* in immunized BALB/c mice was observed in this study, as compared to the control groups. However, on the 18^th^ day after challenge all mice were dead and constructed DNA vaccines only provided partial protection. The data also showed that mice immunized with pSAG1/14-3-3 induced a stronger humoral and cellular immune response and significantly prolonged survival time when compared with pSAG1 or p14-3-3 group. Our results described above are consistent with previous studies showing that mice immunized with a plasmid encoding the SAG1 gene gave effective protection against *T. gondii* infection [29,43]. The major limitation of the current study is the lack of the evaluation of tissue cysts numbers in BALB/c mice. Preventing tissue cyst formation in animals for consumption can help avoid parasite transmission to humans and animals, which is important in the farming industry [12]. Experimental studies showed that DNA vaccination can induce both survival prolongation and brain cyst reduction in rodents [44,45]. An effective and safe vaccine should protect the hosts against *T. gondii* infection and reduce the potential risk of tissue cyst generation in intermediate hosts and development of oocysts in cats.

Protective immunity to *T. gondii* is complex and involves lots of elements of the host immune system, furthermore, it is recognized that this infection in immunocompetent hosts elicits a strong Th1 type immunity response, which is characterized by the generation of parasite specific CD4+ and CD8+ T cells that produce IFN-γ and provide protective immunity against *T. gondii*[46,47]. Successful DNA vaccination is in theory well suited to stimulate effector mechanisms depending on antigen presentation along with major histocompatibility complex class 1 (MHC-1) molecules, which stimulate CD8+ cytotoxic T cells [29,48]. In recent years, most study results have indicated that several parameters affect the immune response generated by the DNA vaccine, and in the design of DNA vaccines the candidate antigen and the eukaryotic expression vector were of great concern. Good vaccine candidates should induce protective cellular Th1 and humoral Th2 responses as well, both at the level of intestinal mucosa (local) and whole organism (systemic) [12]. In the present study, the SAG1 and 14-3-3 protein were selected due to their potential to elicit an immune response, our results also confirmed that SAG1 has been a prime antigen because of its immunodominance and 14-3-3 protein is a novel candidate antigen. In the meantime, the pBudCE4.1 co-expression vector is used to construct the DNA vaccines and contains two promoters for high-level, constitutive, independent expression of two recombinant proteins, which is a good choice for the construction of multi-gene vaccine.

## Conclusions

This study suggested that *T. gondii* 14-3-3 protein can induce humoral and cellular immune responses and was a novel DNA vaccine candidate against toxoplasmosis. The results also indicated that multi-gene vaccines significantly enhanced immune responses and protective efficacy and were superior to the single-gene vaccine. However, no effective vaccine that provided complete protection against a lethal challenge with RH tachyzoites was demonstrated, but DNA vaccines are still considered a good strategy in the control of *T. gondii*. A combination of different antigens in a single formulation should be considered and valued to design an effective and durable vaccine against toxoplasmosis. Future research should mainly focus on multi-gene vaccines, and SAG1 gene is one of the best choices and 14-3-3 protein is promising.

## Competing interests

The authors declare that they have no competing interests.

## Authors’ contributions

SH and XQZ conceived and designed the study, and critically revised the manuscript. MM carried out the experiments and drafted the manuscript. GZ and YB contributed to the revision of the manuscript. HZ, HC, GL and QZ helped carry out various aspects of the experiments and revised the manuscript. All authors read and approved the final manuscript.
